# A comprehensive technology strategy for microbial identification and contamination investigation in the sterile drug manufacturing facility—a case study

**DOI:** 10.3389/fmicb.2024.1327175

**Published:** 2024-02-12

**Authors:** Minghui Song, Qiongqiong Li, Chengzhi Liu, Peien Wang, Feng Qin, Lichun Zhang, Yiling Fan, Hong Shao, Guiliang Chen, Meicheng Yang

**Affiliations:** ^1^NMPA Key Laboratory for Testing Technology of Pharmaceutical Microbiology, Shanghai Institute for Food and Drug Control, Shanghai, China; ^2^Shanghai Quality Inspection and Testing Center for Innovative Biological Products, Shanghai, China; ^3^Hangzhou Digital-Micro Biotech Co., Ltd., Hangzhou, China; ^4^Shanghai SPH New Asia Pharmaceutical Co., Ltd., Shanghai, China; ^5^China State Institute of Pharmaceutical Industry Co., Ltd., Shanghai, China; ^6^Shanghai Center for Drug Evaluation and Inspection, Shanghai, China

**Keywords:** microbial identification, pharmaceutical process, contamination investigation, MALDI-TOF MS, sequencing technology

## Abstract

**Objective:**

A comprehensive strategy for microbial identification and contamination investigation during sterile drug manufacturing was innovatively established in this study, mainly based on MALDI-TOF MS for the identification and complemented by sequencing technology on strain typing.

**Methods:**

It was implemented to monitor the bacterial contamination of a sterile drug manufacturing facility, including its bacterial distribution features and patterns. In three months, two hundred ninety-two samples were collected covering multiple critical components of raw materials, personnel, environment, and production water.

**Results:**

Based on our strategy, the bacterial profile across the production process was determined: 241/292 bacterial identities were obtained, and *Staphylococcus* spp. (40.25%), *Micrococcus* spp.(11.20%), *Bacillus* spp. (8.30%), *Actinobacteria* (5.81%), and *Paenibacillus* spp. (4.56%) are shown to be the most dominant microbial contaminants. With 75.8% species-level and 95.4% genus-level identification capability, MALDI-TOF MS was promising to be a first-line tool for environmental monitoring routine. Furthermore, to determine the source of the most frequently occurring *Staphylococcus cohnii*, which evidenced a widespread presence in the entire process, a more discriminating *S. cohnii* whole-genome SNP typing method was developed to track the transmission routes. Phylogenetic analysis based on SNP results indicated critical environment contamination is highly relevant to personnel flow in this case. The strain typing results provide robust and accurate information for the following risk assessment step and support effective preventive and corrective measures.

**Conclusion:**

In general, the strategy presented in this research will facilitate the development of improved production and environmental control processes for the pharmaceutical industry, and give insights about how to provide more sound and reliable evidence for the optimization of its control program.

## Introduction

Microbial contamination of pharmaceutical products continues to be a severe challenge to drug manufacturing. Drug products, especially high-risk sterile products such as injection and ophthalmic products, can cause irreversible damage once contamination occurs during the production and storage process, in severe cases, detrimental to the patient’s health and even their lives. It was shown that between 2012 and 2019, more than 50% of all the recalls related to drug products registered by the US Food and Drug Administration (FDA) were associated with microbiological incidents (Santos et al., 2018; Jimenez, 2019). The high frequency of recalls indicates inadequate awareness of contamination risk and poor implementation of relevant control programs. Substantial evidence such as warning letters, alert notifications, and failures suggests a direct relationship between the level of environmental control and the product’s final quality (Jimenez, 2007; Sandle, 2012; Jain and Jain, 2018).

Understanding the types and distribution of environmental microflora during drug manufacturing is essential to the company’s in-process surveillance program (Hamdy et al., 2018). Once a relevant microflora database is established, it can be used for investigations of product contamination, environmental excursions, etc. The trend changes in the type and number of predominant species may also signal a weakness in contamination control strategy (CCS) and trigger some preventive and corrective actions (Akers, 1997; Halls, 2004). In addition, the database can also provide further insight into other elements of the CCS, such as the design and evaluation of an effective disinfection scheme (Wu and Liu, 2007). It is crucial to implement a microbial identification and characterization framework as part of an effective microbial contamination control strategy. While the consensus has been established and higher expectations have been placed in the latest regulations and guidance, relatively few studies are associated with the microbial profile in clean pharmaceutical environments (Wu and Liu, 2007; Sandle, 2011; Vijayakumar et al., 2012, 2016; Park et al., 2014). Methodologies employed in those studies range from biochemical methods to genomics, which are either complicated, less accurate, or expensive to promote in industry. A fast, accurate, and cost-effective solution, along with a more rapid and efficient tool to speed up and improve the quality of the analytical results, is highly desirable for implementing a comprehensive monitoring system.

Several methods are available for rapid and automated microorganism identification and typing, generally classified as phenotypic or genotypic techniques (Ferone et al., 2020). Matrix-assisted laser desorption ionization-time of flight mass spectrometry (MALDI-TOF MS), a methodology based on the extraction of protein fingerprint signatures from the whole bacterial cells, is raising interest. MALDI-TOF MS has gained extensive clinical use due to its rapid and accurate bacterial identification capabilities (Bizzini and Greub, 2010; Patel, 2015; Singhal et al., 2015). Previous studies showed that bacterial identification using MALDI-TOF MS was significantly better than conventional biochemical systems. It was concluded that MALDI-TOF MS could be implemented quickly to routinely identify microorganisms in a clinical microbiology laboratory (Schubert and Kostrzewa, 2017). However, the progression in using this technique for bacterial identification in other industries like the pharmaceutical area is very slow, this is due to the limited availability of relevant mass spectral libraries, and the bias of existing libraries more focused on clinical isolates (Andrade et al., 2018).

In addition to understanding the bacterial species profiles of the production environment, early detection of cross-transmissions is also critically important in blocking the spread of microbial contamination and quickly implementing control measures to minimize the damages. This introduced a demand for more discriminating methods of bacterial characterization-strain typing, which can characterize and confirm epidemiological linkage in an area and provide insights into bacterial population dynamics (Scott et al., 2002). Multiple methods based on different phenotypic and genotypic principles have been adopted to decipher epidemiological links to identify reservoirs and routes of transmission (Simar et al., 2021); among these, molecular biology methods with a relatively high discriminatory power such as multi-locus sequence typing (MLST) and pulsed-field gel electrophoresis (PFGE) have been proved to be beneficial for surveillance and contamination investigation (Simar et al., 2021). Strain typing methodologies have recently undergone a paradigm shift as whole-genome sequencing (WGS) has become more accessible to clinical and industry laboratories. WGS provides higher discriminatory power for highly related strains, and it has significant potential for outbreak detection, epidemiological surveillance, and infection control strategies (Miro et al., 2020; Uelze et al., 2020; Simar et al., 2021; Menghwar et al., 2022). The identification of single-nucleotide polymorphisms (SNPs) between bacterial isolates is one of the most commonly used analyses of WGS data and has been widely used as an alternative to MLST and PFGE on several bacterial species, facilitating its use in outbreak investigations (McDonald et al., 2006; Roe et al., 2016; Hoefer et al., 2021).

The objectives of this study were to establish a comprehensive strategy for microbial identification and investigation during the drug manufacturing process. The recovered isolates from a monitoring system covering the entire production were classified using a Bruker MALDI-TOF MS system and 16S rRNA gene sequencing combination approach to determine the predominant bacterial flora distributed in the cleanroom environment, the capability of the current MALDI-TOF MS system and its commercial database to identify pharmaceutical industry-relevant contaminants was evaluated. The bacterial distribution features and patterns were further reviewed, and a more discriminative WGS typing method was used to investigate the transmission routes of the predominant and critical contaminating *Staphylococcus cohnii*. This study intends to provide the pharmaceutical industry with a solution on how to understand the microbial risks of the entire manufacturing process.

## Materials and methods

### Environmental monitoring plan and sampling

From June to August 2018, the environmental monitoring program was carried out in a sterile powder for injection drug manufacturing facility in Shanghai, China. Samples were taken from its two primary production areas: formulation manufacture (MF) and substance manufacture (MS), both areas maintained the 4 grades of cleanrooms. Samples were taken from multiple elements of the production process including finished products, raw materials, personnel, production environment, and water. Different pharmacopeial techniques were used: (a) active air sampling using a volumetric air sampler; (b) passive air sampling using settle plates; (c) swabs for irregular surface sampling; (d) contact plates for flat surfaces; (e) 100 mL membrane filtration onto Reasoner’s 2A Agar medium (R2A, bioMérieux, France) for water for injection. Trypticase Soy Agar medium (TSA, bioMérieux, France) was used for the sampling except for the water samples, and a neutralizer to neutralize the effects of disinfectant residues on surfaces was added when necessary. An intensive sampling strategy was implemented, and the sampling frequencies were set according to the potential microbiological contamination risk assessed for different monitoring items and scopes. In general, settle plates were exposed for the duration of operations and changed after 4 h for grades A and B; active air sampling was conducted for each shift. Both settle plates and active air samples were taken for the grade C area for each batch. For hard surfaces like the product contact area, filling machine, and relevant components, sampling was conducted every shift for grades A and B, and each week for grade C. The collected plates were under 30°C–35°C aerobic incubation for 3–5 days for bacterial growth. The specific sample information is listed in Supplementary Table S1.

### Cultivation and isolation

The sample information was recorded in detail according to the rules interpreted in Supplementary Table S1. ChP chapter <9205> (Chinese Pharmacopoeia Commission, 2020 edition) were referred to detect and isolate microorganisms from the samples. In general, after incubation, the plates with microorganism growth were reserved for determination of the next steps: any growth on the plates collected from the A/B grade areas were subcultured and identified; for bacterial colonies on the plates collected from the C/D grade areas, only representative colonies from the same plate, as determined by colony morphology and microscopic examination, were selected for subsequent subculture and identification. The selected colonies were transferred to TSA medium (bioMérieux, France), incubated under appropriate conditions (30°C–35°C aerobic incubation for 3–5 days), and then examined. The type of colonies on the plate was assessed primarily based on the morphological characteristics and recorded. Distinctive colonies were selected and subcultured onto TSA plates and incubated under 30°C–35°C for 1–3 days to get fresh and purified culture for subsequent preservation, identification, and investigation. The pure cultures were picked and fully suspended into 0.5 mL tryptic soy broth (TSB, bioMérieux, France) vials, and then added same volume of 30% sterile glycerol solution. The cryovials were preserved at −20°C.

### MALDI-TOF MS identification

Preparations of bacterial isolates for MALDI-TOF MS measurement were done as previously described (Croxatto et al., 2012). The on-target extraction preparation method was used to identify all the isolates to establish a standardized rapid routine identification process. Briefly, one fresh colony material was picked and smeared onto a 384 polished steel MSP target (Bruker Daltonik GmbH, Germany) using a toothpick, then one μL of 70% formic acid was added onto the bacterial spot and allowed to air dry afterward add one μL of 10 mg/mL a-cyano-4-hydroxy-cinnamic acid (HCCA, Bruker Daltonik GmbH, Germany) matrix solution (50% acetonitrile; 2.5% trifluoroacetic acid; 47.5% distilled water) and allowed to air dry. The acquisition and analysis of mass spectra were then performed by an Autoflex TOF/TOF mass spectrometer (Bruker Daltonik GmbH, Germany) under the linear positive mode, MALDI Biotyper software package (version Compass) with the reference 7,854 database entries and default parameter settings was used. The Bruker Bacterial Test Standard (BTS) was used for calibration according to the manufacturer’s instructions. Two colony/sample material preparations were analyzed for each isolate, and the higher-scoring identification result was taken.

### 16S rRNA gene sequencing analysis

All isolates preserved after purification from the environmental monitoring plan were sub-cultured on TSA medium at 35°C for 24 h during identification. The whole DNA was extracted using a TAKARA DNA extraction kit (Takara Bio Inc., Shiga, Japan) following the manufacturer’s instructions. Primer 27F (5′-AGTTTGATCMTGGCTC AG-3′) and 1492R (5′-GGTTAC CTTGTTACGACTT-3′) were used for PCR amplification. PCR products were resolved and visualized by electrophoresis in gels containing 1.2% agarose and 0.5 lg/mL ethidium bromide. After that, all the amplified DNA fragments were sequenced commercially in both directions (Sangon Co., Ltd., Shanghai), and the sequence reads were analyzed using Lasergene 7.0 (DNAStar, Madison, WI). A query sequence with 98.65% identity to a reference in GenBank was acceptable for species identification (Kim et al., 2014).

### Whole-genome sequencing *of Staphylococcus cohnii*

Genomic DNAs of all 33 *S. cohnii* isolates were extracted from overnight cultures by using a Wizard genomic DNA purification kit (Promega, Beijing, China) and were subjected to whole-genome sequencing using 150 bp pair-end strategies using the Illumina Miseq platform (Illumina Inc., San Diego, CA, United States). All operations followed standard Illumina protocols. Raw reads were trimmed and assembled to contigs using SPAdes version 3.11. Using FDAARGOS_538(GCA_003956025.1) as a reference genome, single-nucleotide polymorphisms (SNPs) were called by the Gingr online software of Harvestsuite.^1^ The phylogenetic trees were analyzed by parsnp software based on the whole genomes, and the trees were visualized with FigTree software (Version 1.4.3) and Adobe Illustrator software (Version 22.1). Homologous recombination events were detected by Gubbins v3.1.3^2^ and related sites were filtered in the subsequent analysis. After filtering, 172 wgSNPs were obtained (Croucher et al., 2014). Simpson’s index of diversity (SID), a discriminatory index of a microbial typing method, was calculated as previously described (Hunter and Gaston, 1988).

## Results

### The abundance of microorganisms at different processing locations

From June to August 2018, 292 isolates were recovered and purified from the production process of a sterile drug manufacturing facility, including the materials, personnel, environment, and the production water system, respectively. The bacterial contamination distribution is shown in Table 1. The data showed that the largest contamination source was the production environment (212 isolates), occupying more than 70% of all isolates, followed by the water system (39 isolates). Further examination of the contamination distribution in different grade areas indicated that the highest rate (52%) was found in grade C clean areas, with a decreasing tendency to the higher-grade cleanroom. The increased quantity and diversity of contaminants suggested a wide range of contamination sources. Notably, 30 isolates were recovered from the grade A production area, demonstrating the existence of severe contamination within this production environment, and relevant action should be taken urgently.

**Table 1:** The distribution of microorganisms in different locations categorized by number of isolates, genus, and species.

Environmental monitoring objectives		Numbers of isolates		Percentage (%)	Genus	Species
Raw materials		30		10.27	9	19
Personnel		11		3.77	8	8
Production environment	A	212	30	72.60	8	16
	B		71		15	28
	C		111		16	31
Water		39		13.36	14	24
Total		292		100.00	44	94

### Characterization of microflora in the pharmaceutical production environment

The 292 isolates were identified using a multiplex method of MALDI-TOF MS and 16S rRNA gene sequencing. Then, the potential duplicates of the 292 isolates were excluded, combining with the isolation context, growth features, colony morphology and species information. The identification results of the 241 selected isolates using MALDI-TOF MS method are given in Supplementary Table S2. Of these samples, 189 confident results were obtained, including 147 (61.0%) with species-level results (score ≥2.0) and 42 (17.4%) with genus-level results (1.7 ≤ score < 2.0) according to the manufacturer’s threshold interpretation. Non-reliable identification results (score <1.7) were given to the remaining 52 isolates (21.6%).

**Figure 1:**
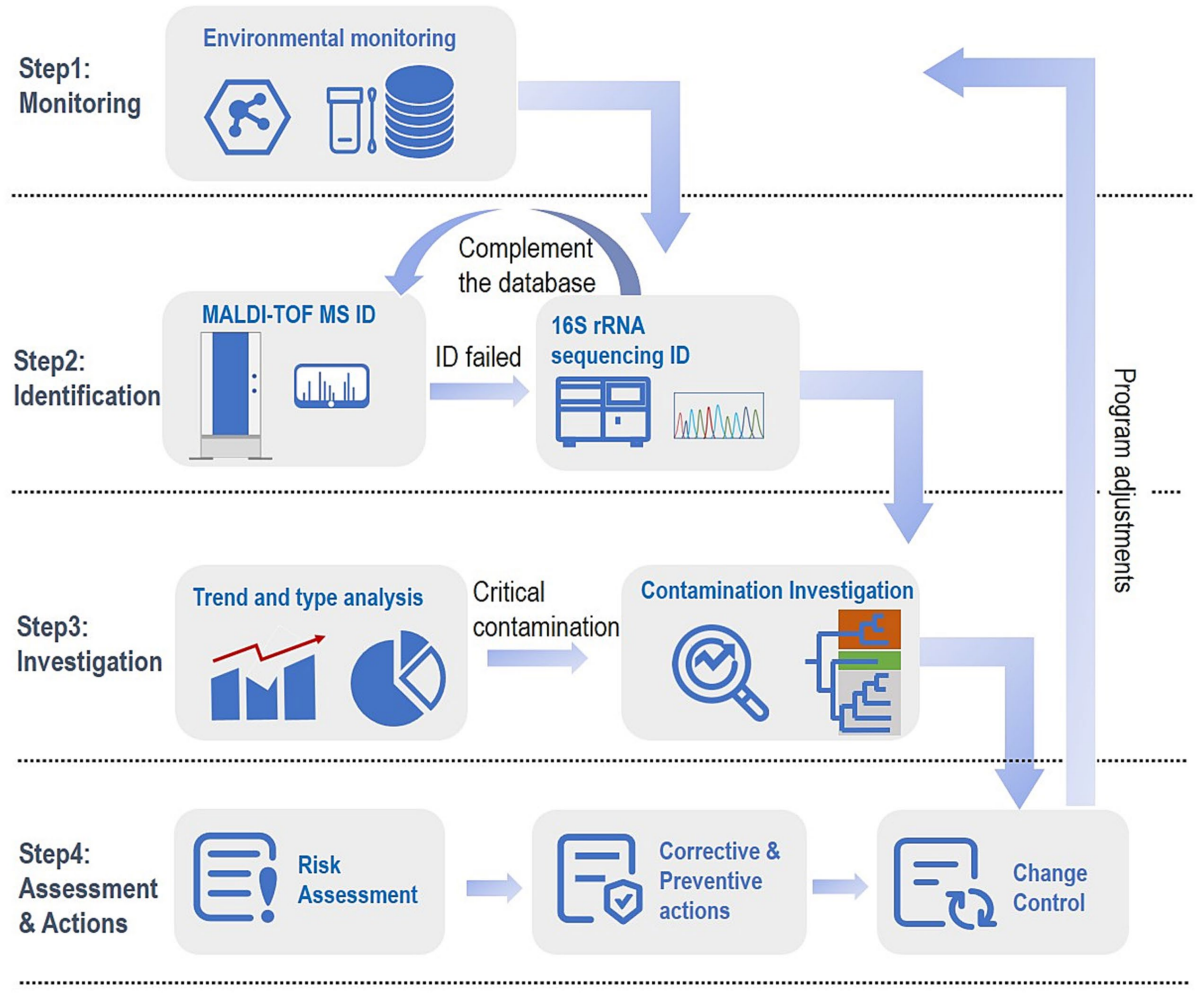
Illustration of a comprehensive monitoring and tracking process. Step 1 Monitoring: A comprehensive monitoring plan is designed based on the production process and facility environment. Step 2 Identification: MALDI-TOF MS is used as the principal identifier, 16S rRNA gene sequencing as the complemented tool for MALDI-failed ID, and confirmed sequencing results are further used to expand the MALDI customized database. Step 3 Investigation: Based on the identification results of step 2, the bacterial profiles of the facility obtained can be used for the trending analysis about the isolation from critical areas (e.g., grade A) or the predominant species; further tracking process will proceed, this process generally involves in a typing methodology with a discriminating power to strain-level. Step 4 Assessment and Actions: After integrating the evidence obtained, a risk assessment process is followed by implementing targeted corrective and preventive measures, completed with the change control.

The total of 94 isolates with low confidence (1.7 ≤ score < 2.0) and no identification results were further subjected to 16S rRNA gene sequencing as the flow illustrated in Figure 1 as complementation. The sequencing details are shown in Supplementary Table S2. Among the 94 unreliable results obtained by MALDI-TOF MS, 79 isolates were identified as species-level by the matching criteria (≥98.65% similarity), and 15 isolates were identified as genus-level. Integrating the results of the whole identification module, it was finally determined that the 241 isolates were from 44 genera and 94 species. With the high-throughput capability, all mass spectrometry identifications can be accomplished within a single batch on the same day.

As shown in Table 2; Supplementary Table S2, comparing the 16 s rRNA gene sequencing identification results with the MALDI-TOF MS commercial database revealed that of the 94 unreliable MALDI-identifies, 46 isolates from 31 species were not covered by the commercial MALDI database. If we remove those out-of-coverages, the identification capacity of MALDI-TOF MS method could reach 75.8% species-level and 95.4% genus-level. The most significant discrepancy impacted by database coverage was with *Bacillus*, where the accuracy of species-level identification improved from 30% to 85.7% by removing non-coverage species from the identification set.

**Table 2:** Evaluation of the identification capability of MALDI-TOF MS for all isolates collected and the dominant contaminated genus in this study.

Genus	MALDI^a^ claimed covered species	Isolates in this study	MALDI not covered	Identification results for all isolates		Identification results only MALDI covered	
		Isolates (species)	Isolates (species)	Species level	Genus level	Species level	Genus level
*Staphylococcus*	45	97 (10)	3 (1)	80.4% (78)	13.4% (13)	83%	13.8%
*Micrococcus*	6	27 (4)	0 (0)	85.2% (23)	14.8% (4)	85.2%	14.8%
*Bacillus*	108	20 (10)	13 (6)	30% (6)	5% (1)	85.7%	14.3%
*Actinobacteria*	39	14 (4)	1 (1)	57.1% (8)	21.4% (3)	61.5%	23.1%
*Paenibacillus*	85	11 (3)	0 (0)	72.7% (8)	27.3% (3)	72.7%	27.3%
In total	2,969	241 (92)	46 (31)	60% (147)	17.4% (42)	75.4%	21.5%

a MALDI here specifically refers to the MALDI system and database version used in this study, as stated in Materials and Methods section.

### The microflora in the pharmaceutical production environment

Of the 241 identified isolates, which represent44 genera and 94 species, the most predominant species/genera (≥3 isolations) are shown in Table 2. As demonstrated, the most significant proportion of genus-level contamination was associated with *Staphylococcus* spp. (40.25%), *Micrococcus* spp. (11.20%), *Bacillus* spp. (8.30%), *Actinobacteria* (5.81%), and *Paenibacillus* spp. (4.56%), the top 5 genera contributing to 70% of all the isolates. The 19 predominant species accounted for 64% of all 94 species isolated, with a distinct predominance of *Staphylococcus* spp., which occupied 9 of the 19 species that comprise most of the microflora, of which *S. cohnii*, *S. epidermidis,* and *S. hominis* were detected more than 10 times. *Micrococcus luteus*, was also shown to be a frequently occurring contaminant (Eissa, 2016).

**Figure 2:**
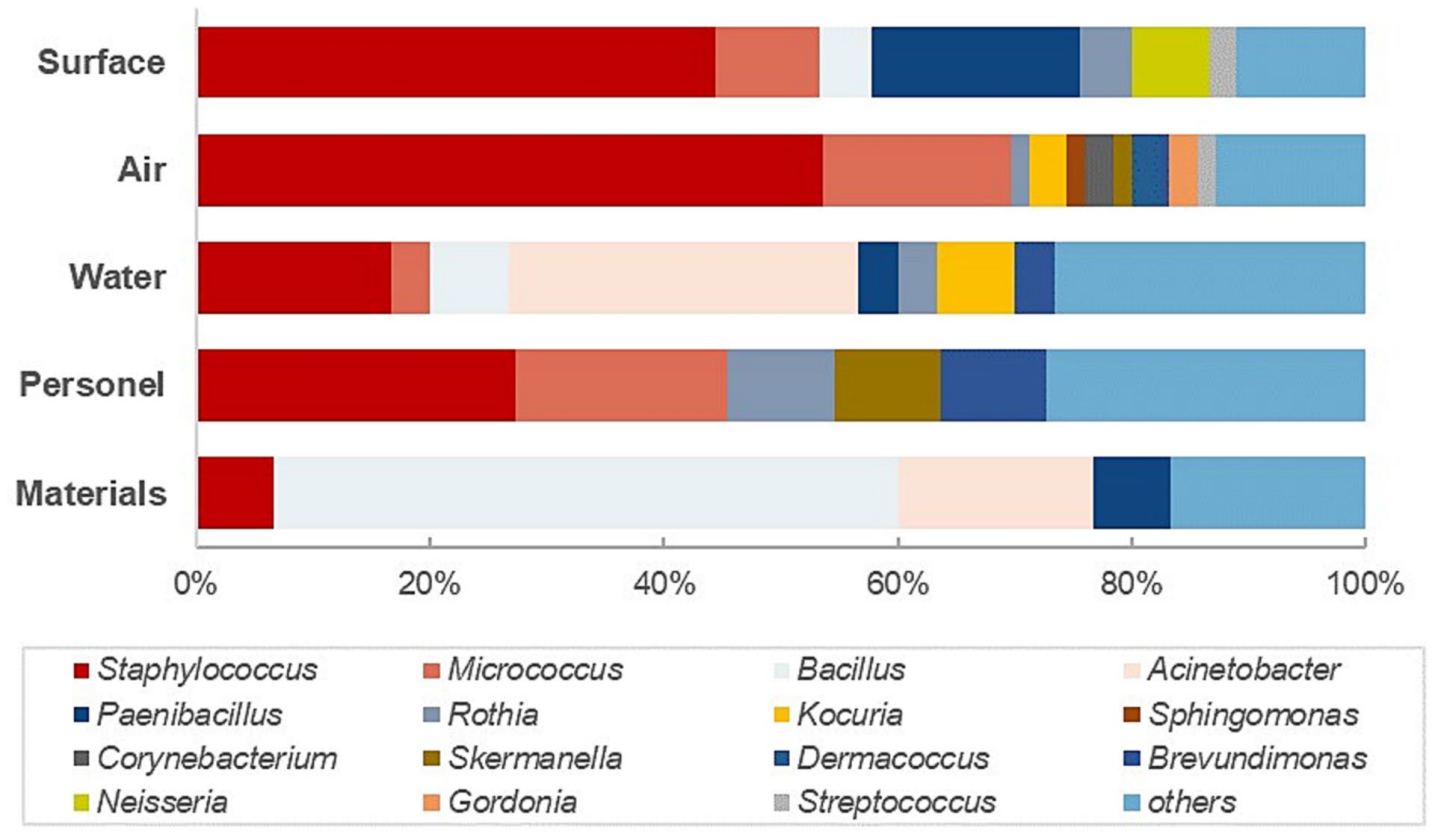
The distribution of the predominant genus in different monitoring locations.

The investigation plan further classified the monitoring objective into airborne (settle plates and volumetric air sampling) and surface flora (contact plates and swabs) for a more specific analysis. As illustrated in Figure 2, the most diverse microflora in quantity and type was airborne flora, covering 26 different genera, with the majority recovered of *Staphylococcus* spp. (69%) and *Micrococcus* spp. (74%). Within the predominant genus listed in Table 3 (≥ three isolations), four genera were only detected from airborne sampling. Unexpectedly, no spore-producing *Bacillus species* was identified from the air samples of this study, which deviates from the conventional understanding that most spores could be transmissible through the air, which could be related to the selection of sampling locations and sampling protocols. The same with the highly abundant detection of Gram-positive cocci in the water system which might be related to inappropriate sampling practices. These are the aspects that need to be improved in the subsequent design and execution of the environmental monitoring protocols. In contrast, 58% of the bacterial spore-forming species were isolated from raw materials and 32% from environmental surfaces, highlighting the critical importance of raw material quality control and transfer disinfection.

**Table 3:** Most commonly occurring species/genera in the pharmaceutical production environment.

Species	Isolation	Percentage
*Staphylococcus cohnii*	33	13.69%
*Staphylococcus epidermidis*	27	11.20%
*Micrococcus luteus*	17	7.05%
*Staphylococcus hominis*	10	4.15%
*Paenibacillus lautus*	9	3.73%
*Staphylococcus haemolyticus*	6	2.49%
*Acinetobacter pittii*	6	2.49%
*Staphylococcus nepalensis*	5	2.07%
*Bacillus aryabhattai*	5	2.07%
*Staphylococcus pasteuri*	4	1.66%
*Staphylococcus capitis*	4	1.66%
*Micrococcus cohnii*	4	1.66%
*Micrococcus antarcticus*	4	1.66%
*Staphylococcus saccharolyticus*	3	1.24%
*Staphylococcus argensis*	3	1.24%
*Rothia amarae*	3	1.24%
*Bacillus paralicheniformis*	3	1.24%
*Bacillus megaterium*	3	1.24%
*Acinetobacter nosocomialis*	3	1.24%
Others	89	36.93%

Genus	Isolation	Percentage
*Staphylococcus*	97	40.25%
*Micrococcus*	27	11.20%
*Bacillus*	20	8.30%
*Acinetobacter*	14	5.81%
*Paenibacillus*	11	4.56%
*Rothia*	6	2.49%
*Kocuria*	4	1.66%
*Sphingomonas*	4	1.66%
*Corynebacterium*	3	1.24%
*Skermanella*	3	1.24%
*Dermacoccus*	3	1.24%
*Brevundimonas*	3	1.24%
*Neisseria*	3	1.24%
*Gordonia*	3	1.24%
*Streptococcus*	3	1.24%
Others	37	15.36%

### Contamination source tracking of *Staphylococcus cohnii* using the WGS typing method

**Figure 3:**
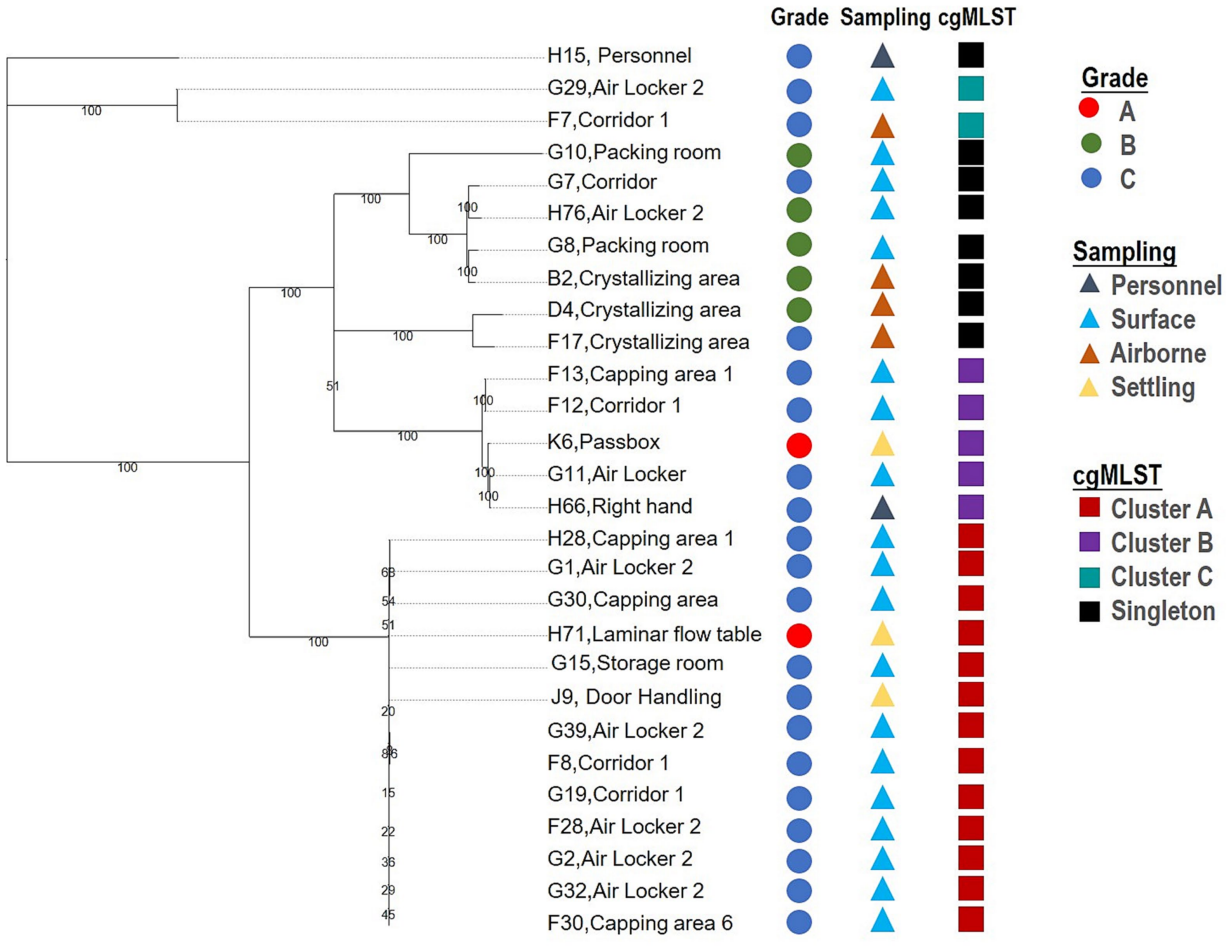
Result of wgSNP typing of the 28 *S. cohnii* contaminated.

As the present results revealed, *Staphylococcus* spp. is the primary microbial contaminant during this drug production process, accounting for 40.25% of the viable contaminant, of which species *S. cohnii* was the most critical one, contributing 33 isolates recovered in this study, the information of the isolates is given in Supplementary Tables S1, S2. It was shown that *S. cohnii* were recovered from grade A (3), grade B (7), grade C (20) environments, personnel (2), as well as raw materials (1), evidencing its widespread presence in the entire process. In the two key areas where the environmental monitoring was conducted, the majority (28/33 isolates) of the recoveries of *S. cohnii* were from the formulation manufacture (MF), and only five were from the substance manufacture (MS). As shown in Figure 3, there were two critical areas (grade A) of contamination presence among the 28 isolates recovered from MF, and these isolates were further subjected to a retrospective investigation.

The contamination investigation of the 28 *S. cohnii* strains was carried out with a *Staphylococcus* whole genome SNP typing method established by our laboratory. The analysis results are shown in Figure 3. Twenty-eight isolates were classified into 11 genotypes (SID = 0.765), with three clusters, A, B, and C containing more than two isolates. The strain H71 which recovered from MF’s critical area grade A—laminar flow cabinet, was classified into clustered A with 13 nearly identical isolates (between 20 and 172 SNPs differences). Strain H71 was closely related to strains recovered from several sampling points located in its grade C background area, i.e., airlocks (G2, G1, G32, G39, F28), capping rooms (H28, G30 F30), corridor (F8, G19), etc., indicating a high probability that the contamination of the grade A surface might originate from the lower-level grade C background area where the transmission occurred either through the material, air or personnel flow, considering its process and isolation level, the latter is more probably. The cluster B suggested a high potential for introducing contamination through poor personnel practice or movement, with strain K6 found in the grade A area being closely related to strain H66 (38 SNPs), which was recovered from the right wrist of work staff.

Unlike the MF area, where a more significant transmission route was observed, the 5 *S. cohnii* strains recovered from the MS production area did not show substantial relevancy to each other, indicating the presence of *S. cohnii* in the area was incidental and associated transmission has not yet occurred (data not shown).

## Discussion

Based on the understanding and assessment of a sterile drug manufacturer’s process and facility, a microbial surveillance scheme in the present study, including monitoring, identification, contamination tracking, and risk assessment, was established. The types and distribution of microflora across the entire production process were examined comprehensively. A total of 292 bacterial isolates of bacteria were recovered within 3 months, with a wide range of sources covering all parts of the production process and different grades of clean areas, prompting a high contamination risk and the urgency to optimize its microbial surveillance program and related prevention measures, such as an adequate cleaning and disinfection program.

In this study, we used MALDI-TOF MS as the first-line identification method, complemented by sequencing, enabling a quick characterization of production environmental flora, and the development of a rapid and effective surveillance scheme. As numerous studies have confirmed that MALDI-TOF MS is a fast, simple, and high-throughput proteomic technique for the identification of a variety of bacterial species, in the present study, where database coverage was taken into consideration, the identification accuracy could be improved to75.8% species-level and 95.4% genus-level, this also adequately confirms MALDI-TOF MS can be utilized as a first-line tool for routine environmental monitoring.

But based on the commercial database, only 77.4% of genus-level identities and 60.0% of species-level identities were obtained; when it comes to the case of *Bacillus* spp., the results are even worse (30% species-level and 35% genus-level), though the commercial database contains over 100 species of *Bacillus*. This result is lower than some evaluation studies from clinics, which can typically achieve 90% or higher species-level identification (Croxatto et al., 2012; Patel, 2015; Schubert and Kostrzewa, 2017), This discrepancy observed may arise from the lack of sufficient reference spectrums in the commercial database of MALDI-TOF MS systems. Even though the species-level may be covered by the database, the diversity of strains may be incomplete, as these isolates are collected from the highly controlled and disinfected clean environments, the strains are more stressed than the clinical collected isolates. Our findings are concordant with Seuylemezian’s et al. (2018) studies on clean rooms of spacecraft, which under more rigorous microbial reduction techniques and the manufacturer-provided database yielded successful identification of only 8% of bacterial isolates. These results demonstrated that commercial MALD-TOF databases need to be enhanced by the inclusion of more isolations from pharmaceutical cleanroom facilities, which requires the developers of MALDI-TOF Mass systems to work more closely with pharmaceutical manufacturers. Meanwhile, because of the differences of MALDI users existing in geographic areas, product types, and production processes, some unique strains may exist in their manufacturing environments, using these strains to establish their own customized reference database is also an effective way to improve the identification capability of the MALDI system.

The increased availability of faster and more accurate analytical methods facilitates a more in-depth understanding of microflora distribution and contamination sources in the pharmaceutical manufacturing environment. For this case, 44 genera and 92 species were classified in 2 months across various locations and grades, indicating relatively high risk and wide-ranging sources of contamination in the production process and environment. In line with previous studies, *Staphylococcus* spp. and *Micrococcus* spp. remained the dominant contaminants, reconfirming that the personnel could be the most significant contributor to cleanroom contamination. The distinct predominance of *Staphylococcus* spp., confirms our concern for CoNS contamination in the pharmaceutical industry (Song et al., 2019). At the same time, the presence of resistant bacterial spores present a considerable challenge to the CCS, particularly the validity of disinfection programs during the pharmaceutical process, further highlighting the imperative for the periodic use of sporicide as regulations are expected.

Whole Genome Sequencing (WGS) is a strain-typing technique under development but is already being increasingly exploited in tracing transmission routes and identifying contamination events that occur in the production-to-patient (Balloux et al., 2018; Simar et al., 2021). With the more comprehensive properties, WGS analysis has the potential to be integrated into and strengthen microbiological risk assessment. In this research, we used whole genome SNP typing method to track the source of the most frequently contaminated species, 28 *S. cohnii* isolates distributed widely across grades A to C. The results demonstrate that the contamination of high-grade areas is most likely introduced by lower-grade air or personnel activities. This case also indicates that species-level identification is inadequate to give definitive tracing evidence under certain scenarios; more discriminatory typing methods at the strain-level are required to confirm or rule out the correlation.

In contrast to the conventional practice when a microbiological environmental monitoring excursion occurs, the unavailability of precise contamination risk information leaves only the option of non-targeted and extensive cleaning and disinfection procedures, which are costly, time-consuming, and compromise production efficiency. The bacterial contamination profiling scheme we established as illustrated in Figure 1, based on substantial species distribution and tracking information, will enable more accurate location of the contamination, integrate more evidence for risk assessment, facilitate the formulation and implementation of more targeted corrective and preventive measures, and provide a more scientific and substantiated basis for the development and refinement of contamination control strategies for the whole manufacturing process and associated environment.

The present study developed a multi-method solution mainly based on MALDI-TOF MS and complemented by sequencing to monitor and track pharmaceutical bacterial contamination. Due to the limitations of the MALDI database and the challenges of sample collection and cultivation, we did not address fungi, especially mold detection in this study. As illustrated in Figure 1, a dynamic, life-cycle approach will be our next research blueprint: leveraging accurate sequencing results for constant complementation of the MALDI-TOF MS database, gradually enhancing MALDI’s identifying capability by creating a custom database, establishing an effective way to reduce the microbial burden and improve the level of aseptic assurance.

## Data availability statement

The original contributions presented in the study are included in the article/Supplementary material, further inquiries can be directed to the corresponding authors.

## Author contributions

MHS: Writing – original draft, Writing – review & editing, Funding acquisition, Methodology, Project administration. QQL: Data curation, Methodology, Writing – review & editing. CZL: Data curation, Methodology, Writing – review & editing, Software. PEW: Data curation, Methodology, Writing – review & editing. FQ: Writing – review & editing. LCZ: Writing – review & editing, Investigation. YLF: Writing – review & editing, Funding acquisition. HS: Writing – review & editing. GLC: Writing – review & editing. MCY: Writing – review & editing, Funding acquisition.

## Footnotes

^1^ http://github.com/marbl/harvest

^2^ https://github.com/nickjcroucher/gubbins
