# Supplementary material for: A comprehensive technology strategy for microbial identification and contamination investigation in the sterile drug manufacturing facility—a case study
Source: Front Microbiol. 2024 Feb 12;15:1327175. doi: 10.3389/fmicb.2024.1327175 (PMC10895062; doi:10.3389/fmicb.2024.1327175)
Supplement: Supplementary file 1 [file Table_1.DOCX]

Supplementary Material

**Supplementary Table 1:** The sample information recovered from pharmaceutical manufacturing.

| **Isolates**  **Number** | **Sample**  **Code** | **Sampling**  **Location** | **Cleanliness Grade**  **C**lassification |
| --- | --- | --- | --- |
| A1 | Ⅵ-W-20180519-3 | Water for Injection | B |
| A2 | Ⅵ-W-8-20180519-2 | Water for Injection | B |
| A3 | Ⅵ-F-20180523 | Crystallization Room | B |
| A4 | Ⅵ-F-20180518-1 | Crystallization Room | B |
| A5 | Ⅵ-F-20180518-4 | Crystallization Room | B |
| A6 | Ⅵ-F-20180518-6 | Crystallization Room | B |
| B1 | Ⅵ-F-20180524 | Distribution Room | B |
| B10 | Ⅵ-M-20180518-1 | Activated Charcoal | N/A |
| B11 | Ⅵ-M-20180520 | Activated Charcoal | N/A |
| B12 | Ⅵ-M-20180520-1 | Activated Charcoal | N/A |
| B14 | Ⅵ-M-20180521 | Activated Charcoal | N/A |
| B2 | Ⅵ-F-20180525 | Crystallization Room | B |
| B3 | Ⅵ-W-20180520 | Water for Injection | B |
| B4 | Ⅵ-W-20180519 | Water for Injection | B |
| B5 | Ⅵ-M-201806 | FDP | N/A |
| B6 | Ⅵ-M-201806 | FDP | N/A |
| B7 | Ⅵ-C-20180518 | Beater Pulverizer | B |
| B8 | Ⅵ-C-2-20180524 | Activated Charcoal | B |
| B9 | Ⅵ-C-20180518 | Electronic Scale | B |
| C1 | Ⅵ-W-20180519-1 | Water for Injection | B |
| C10 | Ⅵ-F-20180519-1 | Crystallization Room | B |
| C11 | Ⅵ-F-20180518-5 | Crystallization Room | B |
| C12 | Ⅵ-F-20180518-2 | Crystallization Room | B |
| C13 | Ⅵ-M-20180519 | FDP | B |
| C3 | Ⅵ-C-20180523 | Trolley | B |
| C4 | Ⅵ-C-20180522 | RABS gloves | A |
| C5 | Ⅵ-M-20180518 | Activated Charcoal | N/A |
| C6 | Ⅵ-F-20180518-3 | Crystallization Room | B |
| C7 | Ⅵ-F-20180518-1 | Crystallization Room | B |
| C9 | Ⅵ-F-20180518-3-1 | Crystallization Room | B |
| D1 | Ⅵ-M-180603-1 | FDP | N/A |
| D10 | Ⅵ-M-20180525 | 0.22μm Filter | B |
| D11 | Ⅵ-M-20180525 | 0.22μm Filter | B |
| D12 | Ⅵ-M-20180525 | 0.22μm Filter | B |
| D13 | Ⅵ-M-20180525 | 0.22μm Filter | B |
| D15 | Ⅵ-M-20180525 | 0.22μm Filter | D |
| D17 | Ⅵ-M-20180605 | Nitrogen Return | B |
| D18 | Ⅵ-M-180306-1 | FDP | N/A |
| D19 | Ⅵ-M-180306-3 | FDP | N/A |
| D2 | Ⅵ-F-20180521-1-1 | Crystallization Room | B |
| D4 | Ⅵ-F-20180521-1-3 | Crystallization Room | B |
| D5 | Ⅵ-F-20180521-2 | Crystallization Room | B |
| D6 | Ⅵ-F-20180520 | Holding Room | B |
| D7 | Ⅵ-F-20180522 | Distribution Room | B |
| D8 | Ⅵ-M-20180525 | 0.22μm Filter | D |
| E1 | Ⅵ-F-20180605-4 | Crystallization Room | B |
| E10 | Ⅵ-W-20180607 | Water for Injection | B |
| E11 | Ⅵ-W-20180605-1 | Water for Injection | B |
| E12 | Ⅵ-W-6-20180605-2 | Water for Injection | B |
| E13 | Ⅵ-F-20180606-1 | Crystallization Room | B |
| E16 | Ⅵ-F-20180605 | Distribution Room | B |
| E2 | Ⅵ-C-20180525 | Trolley | B |
| E3 | Ⅵ-F-20180604 | Distribution Room | B |
| E4 | Ⅵ-C-20180524-2 | RABS gloves | A |
| E5 | Ⅵ-F-20180605-2 | Distribution Room | B |
| E6 | Ⅵ-F-20180605-1 | Distribution Room | B |
| E7 | Ⅵ-20180603-2 | Water for Injection | B |
| E8 | Ⅵ-W-20180603-1 | Water for Injection | B |
| E9 | Ⅵ-W-20180602 | Water for Injection | B |
| F1 | Ⅵ-W-20180615-1 | Water for Injection | B |
| F10 | Ⅰ-W-20180610-1 | Water for Injection | B |
| F11 | Ⅵ-W-20180612-1 | Water for Injection | B |
| F12 | Ⅰ-S-20180611-3 | Corridor 1 | C |
| F13 | Ⅰ-S-20180611-1 | Capping Room1 | C |
| F15 | Ⅰ-S-20180613 | Capping Room1 | C |
| F16 | Ⅰ-S-20180615-3 | Capping Room1 | C |
| F17 | Ⅵ-F-20180607-1 | Crystallization Room | B |
| F18 | Ⅵ-F-20180607-2 | Crystallization Room | B |
| F19 | Ⅵ-F-20180607-3 | Crystallization Room | B |
| F20 | Ⅵ-W-20180609-1 | Water for Injection | B |
| F22 | Ⅵ-W-20180609-2 | Water for Injection | B |
| F24 | Ⅵ-W-1-20180609-5 | Water for Injection | B |
| F25 | Ⅵ-F-20180607-2 | Crystallization Room | B |
| F26 | Ⅰ-S-20180613-3 | Air lock2 | C |
| F27 | Ⅰ-S-20180613-2 | Air lock2 | C |
| F28 | Ⅰ-S-20180613-1 | Air lock2 | C |
| F29 | Ⅰ-S-20180613 | Storeroom1 | C |
| F3 | Ⅵ-F-20180608-1 | Crystallization Room | B |
| F30 | Ⅰ-S-20180611 | Capping Room6 | C |
| F31 | Ⅰ-S-20180611-4 | Air lock2 | C |
| F32 | Ⅰ-S-20180611-2-2 | Air lock2 | C |
| F33 | Ⅵ-F-20180608-1 | Distribution Room | B |
| F34 | Ⅵ-W-20180608-1 | Water for Injection | B |
| F35 | Ⅵ-W-20180608-2 | Water for Injection | B |
| F36 | Ⅰ-S-20180612-8 | Air lock2 | C |
| F37 | Ⅰ-S-20180612-6 | Air lock2 | C |
| F38 | Ⅰ-S-20180612-5 | Air lock2 | C |
| F39 | Ⅰ-S-20180612-4 | Air lock2 | C |
| F4 | Ⅵ-W-20180615-4 | Water for Injection | B |
| F40 | Ⅰ-P-20180611-4 | Garment | C |
| F5 | Ⅰ-S-20180613-1 | Capping Room2 | C |
| F6 | Ⅰ-S-20180612 | Storeroom2 | C |
| F7 | Ⅰ-S-20180615 | Corridor 1 | C |
| F8 | Ⅰ-S-20180611-2 | Corridor 1 | C |
| G1 | Ⅰ-S-20180611-3 | Air lock2 | C |
| G10 | Ⅵ-F-20180607-1 | Distribution Room | B |
| G11 | Ⅰ-S-20180615-8 | Air lock2 | C |
| G12 | Ⅰ-S-20180615-6 | Air lock2 | C |
| G14 | Ⅰ-S-20180611 | Air lock2 | C |
| G15 | Ⅰ-S-20180611 | Storeroom1 | C |
| G16 | Ⅰ-S-20180615-1 | Air lock2 | C |
| G17 | Ⅰ-S-20180615-3 | Corridor 1 | C |
| G18 | Ⅰ-S-20180615-2 | Corridor 1 | C |
| G19 | Ⅰ-S-20180615-1 | Corridor 1 | C |
| G2 | Ⅰ-S-20180611-2-1 | Air lock2 | C |
| G20 | Ⅰ-S-20180615-9 | Corridor 1 | C |
| G21 | Ⅰ-S-20180611-7 | Air lock2 | C |
| G22 | Ⅵ-F-20180608-1 | Distribution Room | B |
| G23 | Ⅰ-S-20180612-8 | Air lock1 | C |
| G24 | Ⅰ-S-20180612-2 | Air lock1 | C |
| G25 | Ⅰ-S-20180612-1 | Air lock1 | C |
| G26 | Ⅵ-M-20180607-3 | Activated Charcoal | N/A |
| G27 | Ⅵ-M-20180607-1 | Activated Charcoal | N/A |
| G28 | Ⅰ-S-20180611-3 | Air lock2 | C |
| G29 | Ⅰ-S-20180611-1 | Air lock2 | C |
| G3 | Ⅰ-S-20180615-2 | Air lock2 | C |
| G30 | Ⅰ-S-20180611-1 | Capping Room2 | C |
| G31 | Ⅰ-S-20180613-3 | Corridor 1 | C |
| G32 | Ⅰ-S-20180611-6-2 | Air lock2 | C |
| G33 | Ⅰ-P-20180613-2 | Left elbow | C |
| G34 | Ⅰ-F-20180609-1 | Holding Room | B |
| G35 | Ⅰ-F-20180609-1 | Holding Room | B |
| G36 | Ⅵ-F-20180608-1 | Crystallization Room | B |
| G38 | Ⅰ-S-20180613-1 | Corridor 1 | C |
| G39 | Ⅰ-S-20180611-2 | Air lock2 | C |
| G4 | Ⅰ-S-20180615-2 | Capping Room1 | C |
| G40 | Ⅰ-P-20180611 | Masks | C |
| G5 | Ⅰ-S-20180615-3 | Air lock2 | C |
| G6 | Ⅰ-S-20180611-5-2 | Air lock2 | C |
| G7 | Ⅰ-S-20180614-1 | Corridor | B |
| G8 | Ⅰ-S-20180614-2 | Distribution Room4 | B |
| G9 | Ⅰ-S-20180614-1 | Distribution Room6 | B |
| H1 | Ⅰ-C-20180612 | Screw Bolt Bracket | A |
| H10 | Ⅵ-W-20180615-2 | Water for Injection | B |
| H11 | Ⅰ-S-20180612-9 | Air lock2 | C |
| H13 | Ⅵ-F-20180607-4 | Distribution Room | B |
| H14 | Ⅰ-P-20180611-3 | Garment | C |
| H15 | Ⅰ-P-20180611-2 | Garment | C |
| H16 | Ⅰ-P-20180611-1 | Garment | C |
| H17 | Ⅵ-F-20180605-3-1 | Crystallization Room | B |
| H19 | Ⅵ-F-20180607-2 | Distribution Room | B |
| H2 | Ⅵ-F-20180604 | Distribution Room | B |
| H20 | Ⅵ-C-20180523-2 | Sealing Machine | A |
| H21 | Ⅰ-P-20180613-3 | Right elbow | C |
| H22 | Ⅰ-S-20180613-4 | Corridor 1 | C |
| H23 | Ⅰ-S-20180613-1 | Storeroom1 | C |
| H25 | Ⅵ-F-20180607-4 | Crystallization Room | B |
| H26 | Ⅵ-F-20180607-1 | Distribution Room | B |
| H28 | Ⅰ-S-20180615-1 | Capping Room1 | C |
| H29 | Ⅰ-S-20180615 | Corridor 1 | C |
| H3 | Ⅵ-C-20180522 | Trolley | B |
| H30 | Ⅰ-S-20180615-5 | Corridor 1 | C |
| H31 | Ⅵ-C-20180521-1 | Rack | B |
| H33 | Ⅵ-C-20180523-1 | Sealing Machine | A |
| H35 | Ⅵ-W-20180609-3-2 | Water for Injection | B |
| H36 | Ⅵ-C-20180524-1 | RABS gloves | A |
| H4 | Ⅰ-S-20180611-1-1 | Air lock2 | C |
| H40 | Ⅵ-W-20180609-3-1 | Water for Injection | B |
| H41 | Ⅵ-F-20180605 | Distribution Room | B |
| H42 | Ⅵ-F-20180605 | Crystallization Room | B |
| H43 | Ⅵ-F-20180604-2 | Distribution Room | B |
| H45 | Ⅵ-M-20180603-1 | Activated Charcoal | N/A |
| H46 | Ⅵ-M-20180605-1 | Activated Charcoal | N/A |
| H47 | Ⅵ-M-20180605-2 | Activated Charcoal | N/A |
| H48 | Ⅵ-M-20180603 | Activated Charcoal | N/A |
| H49 | Ⅰ-S-20180612 | Air lock1 | C |
| H5 | Ⅵ-M-20180605-2 | Activated Charcoal | N/A |
| H50 | Ⅵ-M-20180604-2 | Activated Charcoal | N/A |
| H51 | Ⅰ-S-20180612-5 | Air lock1 | C |
| H52 | Ⅵ-W-20180603 | Water for Injection | B |
| H53 | Ⅵ-M-20180604-3 | Activated Charcoal | N/A |
| H56 | Ⅵ-M-20180607-1 | Activated Charcoal | N/A |
| H58 | Ⅵ-C-20180525 | Sterilizer Surface | B |
| H6 | Ⅵ-M-20180605-1 | Activated Charcoal | N/A |
| H60 | Ⅵ-C-20180525 | RABS gloves | A |
| H61 | Ⅰ-S-20180615-4 | Corridor 1 | C |
| H62 | Ⅵ-F-20180604-1 | Distribution Room | B |
| H63 | Ⅵ-F-20180607-3 | Distribution Room | B |
| H64 | Ⅰ-S-20180611-4 | Air lock2 | C |
| H65 | Ⅰ-S-20180611-5-1 | Air lock2 | C |
| H66 | Ⅰ-P-20180611 | Right wrist | C |
| H67 | Ⅰ-S-20180612-10 | Air lock2 | C |
| H68 | Ⅵ-F-20180605-2 | Crystallization Room | B |
| H69 | Ⅵ-C-20180521-2 | Rack | B |
| H7 | Ⅰ-C-20180614 | Transfer Hatch | A |
| H70 | Ⅰ-C-20180609-1 | Transfer Hatch | A |
| H71 | Ⅰ-C-20180611 | Laminar flow bench | A |
| H72 | Ⅰ-S-20180614-3 | Distribution Room4 | B |
| H73 | Ⅰ-S-20180615-5 | Air lock2 | C |
| H74 | Ⅰ-S-20180614-1 | Distribution Room4 | B |
| H75 | Ⅵ-F-20180607-1 | Crystallization Room | B |
| H76 | Ⅰ-S-20180612-3-1 | Air lock2 | C |
| H77 | Ⅰ-S-20180612-3-2 | Air lock2 | C |
| H78 | Ⅰ-S-20180611-1-3 | Air lock2 | C |
| H8 | Ⅰ-S-20180612-6 | Air lock1 | C |
| H80 | Ⅵ-F-20180607-3 | Distribution Room | B |
| H81 | Ⅰ-S-20180614-1 | Distribution Room5 | B |
| H82 | Ⅰ-C-20180612 | Division part | A |
| H83 | Ⅰ-C-20180613 | Division part | A |
| H84 | Ⅰ-W-20180614-1 | Water for Injection | B |
| H85 | Ⅰ-C-20180614 | Division part | A |
| H86 | Ⅰ-C-20180612 | Oscillator | A |
| H87 | Ⅰ-C-20180615 | Division part | A |
| H88 | Ⅰ-C-20180612 | Division part | A |
| J1 | Ⅰ-C-20180613 | Division part | A |
| J10 | Ⅵ-C-20180610 | RABS Roller | A |
| J11 | Ⅵ-C-20180608-1 | Rack | B |
| J13 | Ⅰ-C-20180609-2 | Transfer Hatch | A |
| J14 | Ⅰ-S-20180612-7 | Air lock2 | C |
| J15 | Ⅰ-S-20180612-2 | Air lock2 | C |
| J16 | Ⅵ-C-20180524-2-1 | RABS gloves | A |
| J17 | Ⅵ-C-20180523-5-2-1 | Sealing Machine | A |
| J18 | Ⅵ-C-20180523-5-1 | Sealing Machine | A |
| J19 | Ⅵ-M-20180604-1 | Activated Charcoal | N/A |
| J2 | Ⅵ-C-20180608 | Electronic Scale | B |
| J3 | Ⅵ-C-20180610 | Single-cone | B |
| J4 | Ⅵ-C-20180610 | Crystallization Reactor | B |
| J6 | Ⅰ-S-20180615-7 | Corridor 1 | C |
| J7 | Ⅰ-S-20180611-1 | Corridor 1 | C |
| J8 | Ⅰ-C-20180612 | Division part | A |
| J9 | Ⅰ-C-20180611 | Laminar flow bench | C |
| K1 | Ⅰ-A-20180610 | Final Product | N/A |
| K10 | Ⅰ-P-20180613-1 | Right elbow | C |
| K12 | Ⅰ-P20180613 | Masks | C |
| K13 | Ⅵ-F-20180606-3 | Crystallization Room | B |
| K14 | Ⅵ-C-20180523-5-2-2 | Sealing Machine | A |
| K15 | Ⅵ-F-20180607-5 | Crystallization Room | B |
| K16 | Ⅰ-S-20180612-4 | Air lock2 | C |
| K17 | Ⅵ-C-20180523-4 | Sealing Machine | A |
| K18 | Ⅰ-S-20180612-1 | Air lock1 | C |
| K19 | Ⅰ-C20180613 | Laminar flow bench | A |
| K2 | Ⅰ-W-20180618 | Water for Injection | B |
| K21 | Ⅰ-C-20180613-1 | Laminar flow bench | A |
| K22 | Ⅵ-C-20180524 | RABS gloves | A |
| K3 | Ⅰ-P-20180613 | Masks | C |
| K4 | Ⅰ-W-20180620-1 | Water for Injection | B |
| K5 | Ⅰ-C-20180612 | Laminar flow bench | A |
| K6 | Ⅰ-C-20180615 | Transfer Hatch | A |
| K7 | Ⅵ-W-20180620-1 | Water for Injection | B |
| K8 | Ⅵ-W-20180620-2 | Water for Injection | B |
| K9 | Ⅰ-C-20180614 | Door handle | C |

The Isolates were numbered according to test batches received by the laboratory

The Sample Types codes are defined as **a-b-c**:

**a:** Production line, I-Formulation Manufacture; VI-Substance Manufacture;

**b:** Monitoring objectives and methods, F-viable air sampling using volumetric air; C- air sampling using settle plates; S- swabs or contact plates of the surface; P- personnel, finger plates or gowning sampling; W-water sample and

**c:** Sampling date;

**FDP**: Fructose 1,6-bisphosphate;

**RABS:** Restricted access barrier systems;

**Supplementary Table 2:** The identification results and MALDI database matching with 16S rRNA gene sequencing information

| **Isolates Number** | **Identification**  **Results** | **MALDI score** | **16s Gene Sequencing Matching** | **Exsting in MALDI database** |
| --- | --- | --- | --- | --- |
| A1 | *Sphingomonas hankookensis* | 1.85 | 98.47 | N |
| A2 | *Providencia vermicola* | 1.85 | 99.86 | Y |
| A3 | *Brevundimonas vesicularis* | 2.18 | 99.18 | Y |
| A4 | *Sphingomonas panni* | 1.93 | 98.55 | Y |
| A5 | *Moraxella osloensis* | 2.18 | 99.65 | Y |
| A6 | *Micrococcus flavus* | 1.96 | 99 | Y |
| B1 | *Micrococcus luteus* | 2.29 | 99.86 | Y |
| B10 | *Priestia aryabhattai* | 1.3 | 100 | N |
| B11 | *Bacillus aryabhattai* | 1.42 | 99.86 | N |
| B12 | *Bacillus paralicheniformis* | 1.17 | 99.86 | N |
| B14 | *Bacillus aryabhattai* | 1.4 | 99.38 | N |
| B2 | *Staphylococcus cohnii* | 2.2 | 99.79 | Y |
| B3 | *Acinetobacter pittii* | 1.7 | 99.79 | Y |
| B4 | *Bacillus velezensis* | 1.43 | 99.79 | N |
| B5 | *Acinetobacter lwoffii* | 2.35 | 99.44 | Y |
| B6 | *Paenibacillus provencensis* | 2.1 | 99.79 | Y |
| B7 | *Staphylococcus epidermidis* | 2.04 | 99.58 | Y |
| B8 | *Staphylococcus hominis* | 1.98 | 99.86 | Y |
| B9 | *Staphylococcus epidermidis* | 2.15 | 100 | Y |
| C1 | *Acinetobacter pittii* | 2.02 | 99.72 | Y |
| C10 | *Staphylococcus hominis* | 2.18 | 99.58 | Y |
| C11 | *Micrococcus antarcticus* | 2.28 | 99.64 | Y |
| C12 | *Dermacoccus abyssi* | 1.26 | 99.79 | N |
| C13 | *Bacillus cereus* | 2.1 | 99.93 | Y |
| C3 | *Staphylococcus epidermidis* | 2.07 | 99.79 | Y |
| C4 | *Staphylococcus epidermidis* | 2.12 | 99.72 | Y |
| C5 | *Paenibacillus* | 1.71 | 98.34 | Y |
| C6 | *Staphylococcus hominis* | 2.18 | 99.51 | Y |
| C7 | *Sphingomonas hankookensis* | 1.52 | 98.32 | N |
| C9 | *Staphylococcus epidermidis* | 2.1 | 99.1 | Y |
| D1 | *Lysinibacillus macroides* | 1.35 | 99.58 | N |
| D10 | *Acinetobacter pittii* | 2.21 | 100 | Y |
| D11 | *Escherichia hermannii* | 2.07 | 99.79 | Y |
| D12 | *Exiguobacterium acetylicum* | 1.3 | 99.79 | N |
| D13 | *Acinetobacter pittii* | 2.04 | 100 | Y |
| D15 | *Acinetobacter pittii* | 2.15 | 100 | Y |
| D17 | *Staphylococcus hominis* | 2.45 | 99.65 | Y |
| D18 | *Ponticoccus gilvus* | 1.27 | 96.55 | N |
| D19 | *Staphylococcus cohnii* | 2.09 | 100 | Y |
| D2 | *Staphylococcus epidermidis* | 2.29 | 100 | Y |
| D4 | *Staphylococcus cohnii* | 1.97 | 99.93 | Y |
| D5 | *Staphylococcus equorum* | 1.97 | 100 | Y |
| D6 | *Staphylococcus hominis* | 2.25 | 99.78 | Y |
| D7 | *Micrococcus luteus* | 1.96 | 98.9 | Y |
| D8 | *Acinetobacter nosocomialis* | 2.01 | 100 | Y |
| E1 | *Corynebacterium singulare* | 1.57 | 97.51 | Y |
| E10 | *Stenotrophomonas maltophilia* | 2.25 | 99.86 | Y |
| E11 | *Acinetobacter pittii* | 1.96 | 99.51 | Y |
| E12 | *Staphylococcus epidermidis* | 2.14 | 99.79 | Y |
| E13 | *Staphylococcus epidermidis* | 2.01 | 99.79 | Y |
| E16 | *Staphylococcus epidermidis* | 1.87 | 99.93 | Y |
| E2 | *Brachybacterium paraconglomeratum* | 1.71 | 99.22 | Y |
| E3 | *Staphylococcus hominis* | 2.07 | 99.58 | Y |
| E4 | *Micrococcus luteus* | 2.05 | 99.35 | Y |
| E5 | *Micrococcus luteus* | 2.18 | 99.35 | Y |
| E6 | *Staphylococcus haemolyticus* | 2.45 | 99.65 | Y |
| E7 | *Pseudomonas geniculata* | 1.16 | 99.65 | N |
| E8 | *Acinetobacter sp* | 2.01 | 99.79 | Y |
| E9 | *Acinetobacter nosocomialis* | 2.06 | 99.86 | Y |
| F1 | *Acinetobacter nosocomialis* | 2.28 | 99.38 | Y |
| F10 | *Staphylococcus hominis* | 2.17 | 99.31 | Y |
| F11 | *Staphylococcus hominis* | 2.14 | 99.24 | Y |
| F12 | *Staphylococcus cohnii* | 2.32 | 99.45 | Y |
| F13 | *Staphylococcus cohnii* | 1.97 | 99.04 | Y |
| F15 | *Staphylococcus epidermidis* | 2.15 | 99.38 | Y |
| F16 | *Staphylococcus argensis* | 1.52 | 98.56 | N |
| F17 | *Staphylococcus cohnii* | 2.26 | 99.11 | Y |
| F18 | *Staphylococcus saccharolyticus* | 1.81 | 97.91 | Y |
| F19 | *Staphylococcus haemolyticus* | 2 | 99.58 | Y |
| F20 | *Acinetobacter oryzae* | 1.32 | 98.74 | N |
| F22 | *Brevundimonas faecalis* | 1.48 | 99.32 | N |
| F24 | *Lysinibacillus boronitolerans* | 1.79 | 98.61 | Y |
| F25 | *Micrococcus luteus* | 2.32 | 99.29 | Y |
| F26 | *Staphylococcus argensis* | 1.24 | 98.97 | N |
| F27 | *Staphylococcus nepalensis* | 1.73 | 99.1 | Y |
| F28 | *Staphylococcus cohnii* | 2.29 | 99.52 | Y |
| F29 | *Staphylococcus nepalensis* | 1.85 | 99.1 | Y |
| F3 | *Micrococcus luteus* | 2.39 | 99.57 | Y |
| F30 | *Staphylococcus cohnii* | 2.31 | 99.45 | Y |
| F31 | *Staphylococcus nepalensis* | 1.62 | 99.09 | Y |
| F32 | *Staphylococcus epidermidis* | 2.27 | 99.45 | Y |
| F33 | *Pseudomonas luteola* | 1.95 | 99.44 | Y |
| F34 | *Pseudochrobactrum saccharolyticum* | 1.4 | 98.99 | N |
| F35 | *Acinetobacter oryzae* | 1.83 | 98.74 | N |
| F36 | *Staphylococcus nepalensis* | 1.63 | 99.23 | Y |
| F37 | *Micrococcus luteus* | 2.1 | 99.5 | Y |
| F38 | *Staphylococcus hominis* | 2.03 | 98.91 | Y |
| F39 | *Staphylococcus epidermidis* | 2.15 | 99.59 | Y |
| F4 | *Rothia amarae* | 2.04 | 99.58 | Y |
| F40 | *Micrococcus luteus* | 2.37 | 99.93 | Y |
| F5 | *Staphylococcus epidermidis* | 2.26 | 99.24 | Y |
| F6 | *Staphylococcus epidermidis* | 2.21 | 99.18 | Y |
| F7 | *Staphylococcus cohnii* | 2.04 | 99.38 | Y |
| F8 | *Staphylococcus cohnii* | 2.18 | 99.18 | Y |
| G1 | *Staphylococcus cohnii* | 2.17 | 99.18 | Y |
| G10 | *Staphylococcus cohnii* | 1.93 | 99.25 | Y |
| G11 | *Staphylococcus cohnii* | 1.96 | 99.45 | Y |
| G12 | *Staphylococcus epidermidis* | 2.21 | 99.24 | Y |
| G14 | *Dermacoccus barathri* | 1.54 | 99.37 | N |
| G15 | *Staphylococcus cohnii* | 2.18 | 99.32 | Y |
| G16 | *Staphylococcus haemolyticus* | 2.07 | 99.17 | Y |
| G17 | *Staphylococcus nepalensis* | 1.95 | 99.23 | Y |
| G18 | *Staphylococcus epidermidis* | 2.01 | 99.11 | Y |
| G19 | *Staphylococcus cohnii* | 2.37 | 99.32 | Y |
| G2 | *Staphylococcus cohnii* | 2.34 | 99.45 | Y |
| G20 | *Roseomonas mucosa* | 1.88 | 99.57 | Y |
| G21 | *Staphylococcus argensis* | 1.52 | 98.46 | N |
| G22 | *Staphylococcus epidermidis* | 2.15 | 99.38 | Y |
| G23 | *Massilia suwonensis* | 1.44 | 99.29 | N |
| G24 | *Kocuria assamensis* | 1.37 | 99.72 | N |
| G25 | *Staphylococcus epidermidis* | 2.13 | 97.32 | Y |
| G26 | *Bacillus megaterium* | 2.17 | 99.17 | Y |
| G27 | *Bacillus aryabhattai* | 1.4 | 99.18 | N |
| G28 | *Micrococcus luteus* | 2.15 | 99.43 | Y |
| G29 | *Staphylococcus cohnii* | 2.62 | 99.04 | Y |
| G3 | *Kocuria assamensis* | 1.27 | 99.93 | N |
| G30 | *Staphylococcus cohnii* | 1.95 | 99.38 | Y |
| G31 | *Rothia aeria* | 2.16 | 99.65 | Y |
| G32 | *Staphylococcus cohnii* | 2.07 | 99.18 | Y |
| G33 | *Massilia timonae* | 2.25 | 98.95 | Y |
| G34 | *Staphylococcus pasteuri* | 2.34 | 99.44 | Y |
| G35 | *Staphylococcus epidermidis* | 2.03 | 99.52 | Y |
| G36 | *Micrococcus antarcticus* | 2.2 | 98.81 | Y |
| G38 | *Neomicrococcus lactis* | 1.71 | 99.78 | Y |
| G39 | *Staphylococcus cohnii* | 2.24 | 99.24 | Y |
| G4 | *Staphylococcus epidermidis* | 2.29 | 98.97 | Y |
| G40 | *Rothia amarae* | 2.32 | 99.51 | Y |
| G5 | *Kocuria marina* | 1.96 | 99.44 | Y |
| G6 | *Staphylococcus epidermidis* | 2.23 | 99.17 | Y |
| G7 | *Staphylococcus cohnii* | 2.2 | 99.31 | Y |
| G8 | *Staphylococcus cohnii* | 2.22 | 99.17 | Y |
| G9 | *Micrococcus cohnii* | 2.01 | 99.71 | Y |
| H1 | *Staphylococcus epidermidis* | 2.63 | 99.93 | Y |
| H10 | *Bacillus cereus* | 2.14 | 100 | Y |
| H11 | *Staphylococcus epidermidis* | 2.26 | 99.93 | Y |
| H13 | *Staphylococcus epidermidis* | 2.11 | 99.93 | Y |
| H14 | *Staphylococcus cohnii* | 2.23 | 100 | Y |
| H15 | *Staphylococcus cohnii* | 2.03 | 100 | Y |
| H16 | *Micrococcus luteus* | 2.23 | 99.86 | Y |
| H17 | *Brevibacterium epidermidis* | 1.44 | 99.28 | N |
| H19 | *Corynebacterium hansenii* | 1.64 | 99.56 | Y |
| H2 | *Micrococcus luteus* | 2.07 | 99.78 | Y |
| H20 | *Staphylococcus hominis* | 2.23 | 99.86 | Y |
| H21 | *Roseomonas mucosa* | 2.01 | 99.71 | Y |
| H22 | *Kocuria indica* | 1.38 | 97.53 | N |
| H23 | *Staphylococcus capitis* | 2.65 | 99.64 | Y |
| H25 | *Micrococcus luteus* | 2.65 | 99.71 | Y |
| H26 | *Micrococcus luteus* | 2.34 | 99.78 | Y |
| H28 | *Staphylococcus cohnii* | 2.17 | 99.38 | Y |
| H29 | *Brachybacterium paraconglomeratum* | 1.99 | 100 | Y |
| H3 | *Zimmermannella bifida* | 1.59 | 97.79 | N |
| H30 | *Dermacoccus nishinomiyaensis* | 2.08 | 97.13 | Y |
| H31 | *Bacillus haynesii* | 1.56 | 97.32 | N |
| H33 | *Rothia aeria* | 1.51 | 99.93 | Y |
| H35 | *Ochrobactrum lupini* | 1.48 | 99.93 | N |
| H36 | *Staphylococcus capitis* | 2.18 | 99.93 | Y |
| H4 | *Staphylococcus pasteuri* | 2.5 | 99.86 | Y |
| H40 | *Sphingomonas koreensis* | 1.61 | 100 | Y |
| H41 | *Staphylococcus haemolyticus* | 2.26 | 99.79 | Y |
| H42 | *Micrococcus luteus* | 2.24 | 99.22 | Y |
| H43 | *Staphylococcus capitis* | 2 | 100 | Y |
| H45 | *Bacillus megaterium* | 2,17 | 99.93 | Y |
| H46 | *Bacillus altitudinis* | 2.26 | 100 | Y |
| H47 | *Bacillus paralicheniformis* | 1.23 | 100 | N |
| H48 | *Bacillus aryabhattai* | 1.2 | 100 | N |
| H49 | *Actinomyces oris* | 2.06 | 99.26 | Y |
| H5 | *Bacillus siamensis* | 1.41 | 99.86 | N |
| H50 | *Bacillus siamensis* | 1.23 | 99.93 | N |
| H51 | *Skermanella rosea* | 1.38 | 98.51 | N |
| H52 | *Acinetobacter johnsonii* | 2.24 | 99.28 | Y |
| H53 | *Bacillus toyonensis* | 1.12 | 99.93 | N |
| H56 | *Bacillus megaterium* | 2.06 | 100 | Y |
| H58 | *Staphylococcus cohnii* | 2.03 | 100 | Y |
| H6 | *Bacillus aryabhattai* | 1.17 | 100 | N |
| H60 | *Staphylococcus cohnii* | 2.21 | 100 | Y |
| H61 | *Microbacterium oxydans* | 2.04 | 99.85 | Y |
| H62 | *Micrococcus flavus* | 2.14 | 97.16 | Y |
| H63 | *Micrococcus luteus* | 2.17 | 99.78 | Y |
| H64 | *Janibacter hoylei* | 2.22 | 99.93 | Y |
| H65 | *Gordonia bronchialis* | 1.85 | 100 | Y |
| H66 | *Staphylococcus cohnii* | 1.9 | 100 | Y |
| H67 | *Streptococcus salivarius* | 2.08 | 100 | Y |
| H68 | *Staphylococcus haemolyticus* | 2.14 | 99.93 | Y |
| H69 | *Bacillus licheniformis* | 1.86 | 97.19 | Y |
| H7 | *Staphylococcus saccharolyticus* | 1.66 | 100 | Y |
| H70 | *Macrococcus caseolyticus* | 1.26 | 98.93 | Y |
| H71 | *Staphylococcus cohnii* | 2.34 | 99.93 | Y |
| H72 | *Jeotgalicoccus marinus* | 1.34 | 99.93 | N |
| H73 | *Gordonia bronchialis* | 1.71 | 100 | Y |
| H74 | *Micrococcus cohnii* | 1.93 | 99.93 | Y |
| H75 | *Dermabacter hominis* | 1.79 | 99.93 | Y |
| H76 | *Staphylococcus cohnii* | 2.18 | 100 | Y |
| H77 | *Staphylococcus cohnii* | 2.06 | 100 | Y |
| H78 | *Rothia amarae* | 2.14 | 99.78 | Y |
| H8 | *Staphylococcus epidermidis* | 2.27 | 99.93 | Y |
| H80 | *Micrococcus antarcticus* | 2.28 | 99.64 | Y |
| H81 | *Micrococcus cohnii* | 2.1 | 100 | Y |
| H82 | *Paenibacillus lautus* | 2 | 99.26 | Y |
| H83 | *Paenibacillus lautus* | 1.99 | 99.26 | Y |
| H84 | *Paenibacillus lautus* | 2.32 | 99.26 | Y |
| H85 | *Paenibacillus lautus* | 2.02 | 99.26 | Y |
| H86 | *Paenibacillus lautus* | 2.13 | 99.26 | Y |
| H87 | *Paenibacillus lautus* | 2.22 | 99.26 | Y |
| H88 | *Paenibacillus lautus* | 1.97 | 97.54 | Y |
| J1 | *Paenibacillus lautus* | 2 | 99.07 | Y |
| J10 | *Staphylococcus epidermidis* | 2.36 | 99.11 | Y |
| J11 | *Staphylococcus epidermidis* | 2.21 | 99.38 | Y |
| J13 | *Micrococcus luteus* | 2.43 | 99.57 | Y |
| J14 | *Kytococcus sedentarius* | 1.98 | 98.87 | Y |
| J15 | *Gordonia sputi* | 2.08 | 99.09 | Y |
| J16 | *Neisseria flava* | 1.47 | 99.63 | N |
| J17 | *Rothia dentocariosa* | 2.17 | 98.95 | Y |
| J18 | *Neisseria flava* | 1.41 | 99.63 | N |
| J19 | *Bacillus paralicheniformis* | 1.13 | 99.93 | N |
| J2 | *Staphylococcus saccharolyticus* | 2.07 | 99.1 | Y |
| J3 | *Micrococcus cohnii* | 1.92 | 99.71 | Y |
| J4 | *Staphylococcus pasteuri* | 2.27 | 99.22 | Y |
| J6 | *Mariniluteicoccus endophyticus* | 1.82 | 99.22 | N |
| J7 | *Janibacter hoylei* | 2.26 | 99.02 | Y |
| J8 | *Paenibacillus lautus* | 2.11 | 98.79 | Y |
| J9 | *Staphylococcus cohnii* | 2.39 | 99.38 | Y |
| K1 | *Brevibacillus reuszeri* | 1.93 | 99.72 | Y |
| K10 | *Skermanella aerolata* | 1.49 | 99.85 | N |
| K12 | *Brevundimonas vesicularis* | 2.33 | 98.32 | Y |
| K13 | *Luteimonas arsenica* | 1.38 | 96.27 | N |
| K14 | *Streptococcus sanguinis* | 2.12 | 98.86 | Y |
| K15 | *Corynebacterium tuberculostearicum* | 1.74 | 97.18 | Y |
| K16 | *Streptococcus sp* | 1.97 | 98.69 | Y |
| K17 | *Neisseria subflava* | 1.6 | 99.88 | Y |
| K18 | *Skermanella aerolata* | 1.16 | 99.93 | N |
| K19 | *Brevibacterium casei* | 2.2 | 98.87 | Y |
| K2 | *Quadrisphaera granulorum* | 1.34 | 95.71 | N |
| K21 | *Staphylococcus caprae* | 2.26 | 99.31 | Y |
| K22 | *Staphylococcus capitis* | 2.18 | 99.31 | Y |
| K3 | *Williamsia marianensis* | 1.24 | 99.71 | N |
| K4 | *Micrococcus luteus* | 2.62 | 99.5 | Y |
| K5 | *Micrococcus antarcticus* | 2.24 | 99.44 | Y |
| K6 | *Staphylococcus cohnii* | 2.27 | 99.45 | Y |
| K7 | *Staphylococcus pasteuri* | 2.28 | 99.3 | Y |
| K8 | *Staphylococcus haemolyticus* | 2.13 | 95.47 | Y |
| K9 | *Microbacterium lacticum* | 2.28 | 98.88 | Y |

**Light grey:** Genus-level identification results of 16s rRNA gene sequencing.

**Dark grey**: No reliable identification results of 16s rRNA gene sequencing.
